# Leprosy

**Published:** 1892-06

**Authors:** 


*Leprosy.* By GEORGE THIN, M.D. Pp. xii., 280. London: PERCIVAL & CO. 1891.

The author of this book systematises the knowledge hitherto acquired in relation to the bacillus lepræ, its relations to the pathological changes peculiar to leprosy, and its important bearings on the etiology of the disease. All the evidence tends to show that "the more complete the investigation into the condition of the nerves, the more certain it becomes that the pathology of anæsthetic leprosy has to do with a special morbid process in the nerves, that this process is characterised by inflammation and consequent destruction, and that the cause of the morbid change is to be found solely and simply in the presence and growth of the bacillus lepræ." It follows that "there are very strong grounds for believing that it is contagious in the same sense in which syphilis and tuberculosis are contagious." Much evidence on this question is adduced, and the author naturally states "that there is at present no means of explaining the existence of the bacillus lepræ—the real cause of the disease—in the body of the leper, except by the assumption that he has acquired the parasite directly or indirectly from the body of another leper."

As regards the etiology of the disease, there is not a single fact to show that the poison of leprosy has been once conveyed by anything that a leper has swallowed. The bacillus lepræ is believed to be an exclusive parasite of man; but otherwise there is a close analogy between it and that of tubercle. They both may gain admission into the human frame without leaving any trace at the point of entrance: they both have an uncertain period of incubation: they both may develop rapidly or very slowly; and in both instances, once admission and development have taken place, the complete extinction of the parasite, if it ever occurs, does so with extreme rarity, although in both instances the parasite may be limited to certain isolated parts of the body, and may live very quiescent over a long period of years, the death of the patient in process of time probably taking place from some other disease.

The book shows that, if medicine can do little for the unfortunate leper, medical knowledge can show peoples and governments how, in leper countries, healthy persons may escape the disease.

